# Development of a Spontaneous Liver Disease Resembling Autoimmune Hepatitis in Mice Lacking Tyro3, Axl and Mer Receptor Tyrosine Kinases

**DOI:** 10.1371/journal.pone.0066604

**Published:** 2013-06-17

**Authors:** Nan Qi, Peipei Liu, Yue Zhang, Hui Wu, Yongmei Chen, Daishu Han

**Affiliations:** 1 Department of Cell Biology, Institute of Basic Medical Sciences, Chinese Academy of Medical Sciences, School of Basic Medicine, Peking Union Medical College, Beijing, China; 2 Department of Biochemistry and Molecular Biology, School of Basic Medicine, Anhui Medical University, Hefei, China; 3 Department of Pathology, Navy General Hospital, Beijing, China; University of Chicago, United States of America

## Abstract

Autoimmune hepatitis (AIH) is a severe type of chronic liver disease. The lack of appropriate animal models has resulted in a limited understanding regarding the etiology of AIH. Here, we demonstrated that mice deficient in Tyro3, Axl and Mer (TAM) receptor tyrosine kinases (RTKs) developed persistent inflammatory liver damage resembling AIH. Tyro3^−/−^Axl^−/−^Mer^−/−^ triple mutant (TAM^−/−^) mice exhibited chronic hepatitis, manifested by progressive appearance of interface hepatitis, immune cell infiltrations and elevated inflammatory cytokine levels in the liver. Accordingly, increased levels of transaminases were observed. Moreover, characteristic autoantibodies and high levels of plasma immunoglobulin G for AIH were detected as TAM^−/−^ mice aged. Finally, we provided evidence that the liver damage in TAM^−/−^ mice mainly result from bone marrow-derived cells and could be rescued by transplantation of WT bone marrow cells. Results suggest that TAM RTKs play an important role in maintaining immune tolerance of the liver.

## Introduction

Although the liver is an immunoprivileged site, autoimmune liver diseases, such as autoimmune hepatitis (AIH), develop globally in diverse ethnic groups [1]. AIH is a progressive inflammatory liver disorder that is characterized histologically by interface hepatitis and serologically by high levels of transaminases and the presence of non-organ specific autoantibodies. Two AIH types have been defined according to the autoantibody profile: type 1 (AIH-1) is positive for anti-nuclear antibody (ANA) and/or smooth muscle antibody (SMA), and type 2 (AIH-2) is positive for anti-liver kidney microsomal type 1 (anti-LKM1) antibody [2]. Children and adults of any age may develop AIH [3], [4]. AIH, which is often diagnosed late in its progression, usually results in severe consequences for the patients. Unfortunately, the etiology of the disease and the mechanisms leading to liver damage in AIH are poorly understood. One reason for this limited understanding regarding the etiology of AIH is the absence of reliable animal models. Several mouse models of AIH have been described [5], [6], [7], [8]. However, none of these models show persistent autoimmune liver damage [9]. Studies on immune reactivity against the liver have indicated that self-reactive lymphocytes alone are not sufficient for disease induction without additional inflammatory signals, namely “liver tolerance” [10]. However, upregulation of costimulatory factors in the liver during pathogen-induced inflammation can break this tolerance [11]. Activation of toll-like receptors (TLRs) is engaged to break the immuno-tolerant status of the liver and convert autoreactivity into overt AIH [12].

TLRs belong to a family of pattern recognition receptors that recognize pathogen-associated molecular patterns (PAMPs) from microbes to induce production of pro-inflammatory cytokines for the initial host defense against pathogens [13]. Endogenous components derived from dying host cells, termed damage-associated molecular patterns (DAMPs), can also activate TLRs [14]. Various TLRs are expressed and regulate innate immune responses in the liver [15], [16]. TLR signaling must be tightly controlled for the homeostasis of immune responses, because unrestrained TLR activation generates a chronic inflammatory milieu that can lead to the development of autoimmune diseases [17], [18]. Several mechanisms for the negative regulation of TLR signaling have been identified [19], [20].

TAM subfamily of receptor tyrosine kinases (RTKs) contains three members: Tyro3, Axl and Mer [21]. Two highly similar vitamin K-dependent proteins, product of growth arrest-specific gene 6 (Gas6) and Protein S (ProS, a blood anticoagulant cofactor), are the common ligands of TAM RTKs [22]. Gene knockout studies have provided directly insights into the physiologic functions of TAM RTKs. TAM RTK triple mutant (TAM^−/−^) mice displayed multiple defects in the immune, neuro, reproductive and hematopoietic systems [23], [24], [25], [26], [27]. One of the most prominent functions of TAM RTKs is the negative regulation of innate immune response via inhibition of TLR signaling [28], [29].

In the present study, we demonstrate that TAM RTKs are required for the immune tolerance of the liver, and loss of these receptors results in progressive inflammatory liver damage resembling AIH. The finding fits the previous reports that TAM RTKs inhibit inflammatory response.

## Materials and Methods

### Animals

TAM RTK mutant mice were kindly provided by Dr. Qingxian Lu (Salk Institute for Biological Studies, La Jolla, CA), and were progenies of the original colony with a genetic background of 50% 129/SV × 50% C57BL/6. Wild-type (WT) control mice were the littermates of the mutant mice. The mice were inbred in pathogen-free conditions, and had free access to food and water. Animal study protocol was reviewed and approved by the Institutional Animal Care and Use Committee of Peking Union Medical College Hospital, China [the permit number: SCXK (Jing) 2007-0001]. Female mice were used in this study. All efforts were made to minimize suffering.

### Antibodies

Rat anti-Axl (MAB854), anti-Mer (MAB591 and anti-Tyro3 (MAB759) antibodies were purchased from R&D Systems (Minneapolis, MN). Goat anti-Gas6 (sc-1936), anti-protein S (sc-27027), rabbit anti-NF-κB P65 (sc-372) and anti-phospho-NF-κB P65 (sc-33020) antibodies were purchased from Santa Cruz Biotechnology (Santa Cruz, CA). Rabbit anti-F4/80 (ab6640) antibody was purchased from Abcam (Cambridge, MA). Anti-ED1 (MCA341R) and anti-ED2 (MCA342R) antibodies were purchased from Serotec (Kidlington, UK). Rabbit anti-IRF3 (no. 4302) and anti-phospho-IRF3 (no. 4947) antibodies were purchased from Cell Signaling Technology (Beverly, MA). FITC-conjugated anti-CD4 (557307), anti-CD8 (553034) and anti-B220 (553091) antibodies were purchased from BD bioscience (San Jose, CA).

### Transaminase activity assay

Peripheral blood was collected from the tail vena of mice. Activities of serum alanine aminotransferase (ALT) and aspartate aminotransferase (AST) were measured using ALT and AST enzymatic assay kits (Rongsheng Co., Shanghai, China) following the supplier′s protocol.

### Histological analysis and immunohistochemistry

For histological analysis, the livers were removed from mice and embedded in paraffin. A series of sections (4 µm thick) were cut, and stained with hematoxylin and eosin (H&E).

Immunohistochemistry was performed based on previous description [30]. Briefly, the frozen liver sections (8 µm thick) were incubated with the primary antibodies F4/80, ED1 or ED2 for 60 min at room temperature. The sections were then incubated with the appropriate biotinylated secondary antibodies (Zhongshan Biotechnology Co., Beijing, China) for 60 min, followed by incubation with streptavidin-peroxidase complex for 30 min. Peroxidase binding sites were demonstrated by diaminobenzidine method.

### Immunofluorescence staining

Indirect IF staining was used to detect serum autoantibodies. Hepa1-6 cells cultured on Lab-Tek chamber slides (Nunc, Naperville, IL) were fixed with cold methanol at -20°C for 3 min. After blocking with 10% normal goat serum in PBS at room temperature for 1 h, the cells were incubated with sera obtained from TAM^−/−^ and WT mice at 37°C for 1 h, followed by incubation with fluorescent isothiocyanate (FITC)-conjugated goat anti-mouse immunoglobulin G (IgG) (Zhongshan) for 30 min.

Direct IF staining was performed to examine lymphocytes in the liver. The frozen liver sections were incubated with FITC-conjugated antibodies against B220, CD4 or CD8 for 90 min at room temperature. After rinsing with PBS, the sections were mounted with Canada balsam (Sigma, St. Louis, MO) for observation under a fluorescence microscope (IX71, Olympus).

### Isolation and analysis of lymphocytes from the liver

Lymphocytes were isolated from the liver based on previous description [31]. Briefly, mice were anesthetized with 10 ml 1× PBS (pH 7.0) from the portal vein. The liver was cut into ∼ 1 mm^3^ pieces using a surgical scissor, then were treated with 0.5 mg/ml collagenase type 1 (Sigma) at room temperature for 20 min with gentle pipetting. The suspensions were filtered through 80-µm copper meshes. The cells were separated in 70% percoll solution (Sigma) according to manufacturer′s instructions. After washing twice with PBS, the cells were labeled with FITC-conjugated antibodies against CD4, CD8, or B220, and subsequently analyzed using FACSCanto flow cytometer (BD Biosciences, San Jose, CA).

### Autoantibody detection

The liver was lysed by freezing and grinding. Cytokine concentration in the supernatant was measured using ELISA kits (eBioscience, San Diego, CA). Serum autoantibodies against actin and total plasma IgG were determined using Quanta Lite^TM^ ELISA (INOVA Diagnostic, Inc, San Diego, CA), in accordance with manufacturer′s instructions. The titers of serum autoantibodies against SMA were detected using microscope slides (Beijing EIAab Science Co. Beijing, China) according to manufacturer′s instructions.

### Real-time RT-PCR

Total RNA was isolated from the liver and cells using TRIzol reagent (Invitrogen, Carlsbad, CA) according to the manufacturer′s instructions. RNA (1 µg) was reverse-transcribed into cDNA using Moloney murine leukemia virus reverse transcriptase and random hexamer primers (Promega, Madison, WI). PCR was performed using Power SYBR Green PCR master mix kit (Applied Biosystems, Foster City, CA) in an ABI PRISM 7300 real-time cycler (Applied Biosystems). The relative mRNA levels of target genes were normalized to β-actin. The primers for PCR are listed in Table 1.

**Table 1:** Primers used for real-time RT-PCRs.

	Primer pairs (5′-3′)	
Target genes	Forward	Reverse
IFN-α	GACCTCCACCAGCAGCTCAA	ACCCCCACCTGCTGCAT
IFN-β	GACGTGGGAGATGTCCTCAAC	GGTACCTTTGCACCCTCCAGTA
TNF-α	CATCTTCTCAAAATTCGAGTGACAA	TGGGAGTAGACAAGGTACAACCC
IL-1β	CAACCAACAAGTGATATTCTCCATG	GATCCACACTCTCCAGCTGCA
IL-6	GAGGATACCACTCCCAACAGACC	AAGTGCATCATCGTTGTTCATACA
*β*-actin	GAAATCGTGCGTGACATCAAAG	TGTAGTTTCATGGATGCCACAG

### Western blotting

Tissues or cells were lysed using RIPA lysis buffer (50 mM Tris-HCl, 150 mM NaCl, 0.1% sodium dodecyl sulfate (SDS), 0.5% sodium deoxycholate 1% Triton X-100, 2 mM EDTA, 1 mM dithiothreitol (DTT), pH 7.4). Supernatants were separated by SDS-PAGE and subsequently electrotransferred onto polyvinylidene difluoride membrane (Millipore, Bedford, MA). The membranes were incubated with primary antibodies at 4°C overnight, followed by incubation with the appropriate peroxidase-conjugated second antibodies at room temperature for 1 h. Antigen- antibody complexes were visualized using an enhanced chemiluminescence detection kit (Zhongshan).

### Isolation of mouse parenchymal and nonparenchymal liver cells

Primary parenchymal hepatocytes were isolated as described previously [32]. Briefly, the livers from 10-week-old mice were perfused and digested using 0.05% collagenase IV (Sigma) for 10 min. The liver cell suspension was separated by natural sedimentation once and centrifugation twice at 30 g for 5 min. The parenchymal hepatocytes were recovered from the cell pellets. The fraction of cells in supernatant contained mainly nonparenchymal liver cells including Kupffer cells and sinusoidal endothelial cells, which were separated via density centrifugation on Percoll gradients at 800 g for 15 min based on procedures described previously [33].

### Bone marrow transplantation

Bone marrow cells were collected from donor mice at age 2 months. WT and TAM^−/−^ recipients at 6 months age were lethally irradiated with a single dose of 8.5 Gy ^60^Co and injected with bone marrow cells (5×10^6^ cells each mouse) from donors through tail vein. At 6 months after transplantation, the engrafted mice were analyzed.

### Statistical analyses

Data are presented as mean ± SEM for *n* given samples. Student’s *t*-test was used to determine statistical significance for all comparisons. Calculations were performed with SPSS version 11.0 statistical software (SPSS Inc., Chicago, IL). *P*<0.05 was considered significant.

## Results

### Persistent liver damage in TAM^−/−^ mice

**Figure 1:**
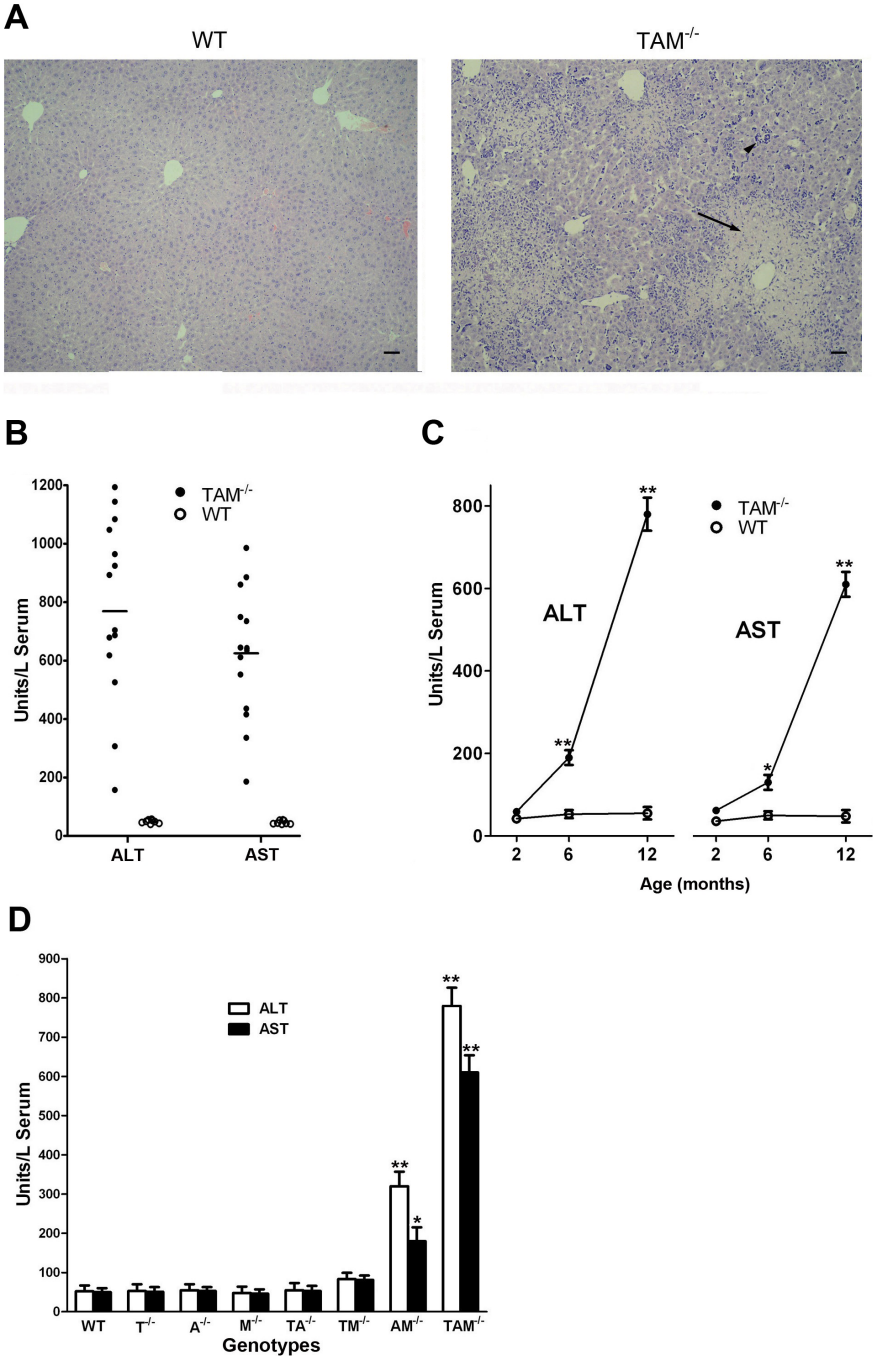
Liver damage in TAM^−/−^ mice. (A) Representative images of H&E stained paraffin-embedded liver sections of 12-month-old WT and TAM^−/−^ female mice. Scale bar  = 10 µm. (B) Serum ALT and AST levels in 12-month-old mice. Each circle represents unit of ALT or AST in sera of an individual mouse. (C) Serum ALT and AST levels in mice at the indicated ages. Note that the livers were progressively damaged as TAM^−/−^ mice aged. (D) Serum ALT and AST levels in 12-month-old mice mutant singly, doubly, and triply for TAM RTKs. T, A, and M represent Tyro3, Axl and Mer, respectively. Data are mean ± SEM, n≥10. *P<0.05, **P<0.01.

Based on histological analysis, a phenotype of chronic hepatitis was found in TAM RTK triple mutant (Tyro3^−/−^Axl^−/−^Mer^−/−^, TAM^−/−^) mice. Severe portal inflammation with piecemeal necrosis and cellular infiltrations into parenchymal regions were frequently observed as TAM^−/−^ mice aged (Figure 1A). Accordingly, increased ALT and AST activities were detected in the sera of TAM^−/−^ mice (Figure 1B). Although a great variance in ATL and AST levels was observed in different mice, all 14 female TAM^−/−^ mice at age 12 months exhibited significant high serum ALT and AST activities compared to age-matched WT controls (Figure 1B). Mean values of ALT and AST activities reached 781 and 653 U/liter serum respectively, which were approximately 15-fold that of WT controls. The liver damage occurred progressively as mice aged. At age 2 months, we detected only basal ALT and AST activities in WT and TAM^−/−^ mice. However, ALT and AST levels were significantly increased at age 6 months and sharply elevated at 12 months in TAM^−/−^ mice (Figure 1C).

The liver damage exhibited a gene dosage effect in TAM RTK mutant series. The increased ALT and AST levels were not observed in mouse single mutants for individual TAM RTKs (Tyro3^−/−^, Axl^−/−^, and Mer^−/−^) and double mutants for Tyro3^−/−^/Axl^−/−^ and Tyro3^−/−^/Mer^−/−^. However, double mutant Axl^−/−^/Mer^−/−^ mice had significantly increased ALT and AST levels compared to WT controls. The most severe liver damage appeared in TAM^−/−^ mice (Figure 1D).

### Immune cell infiltrations in the liver

**Figure 2:**
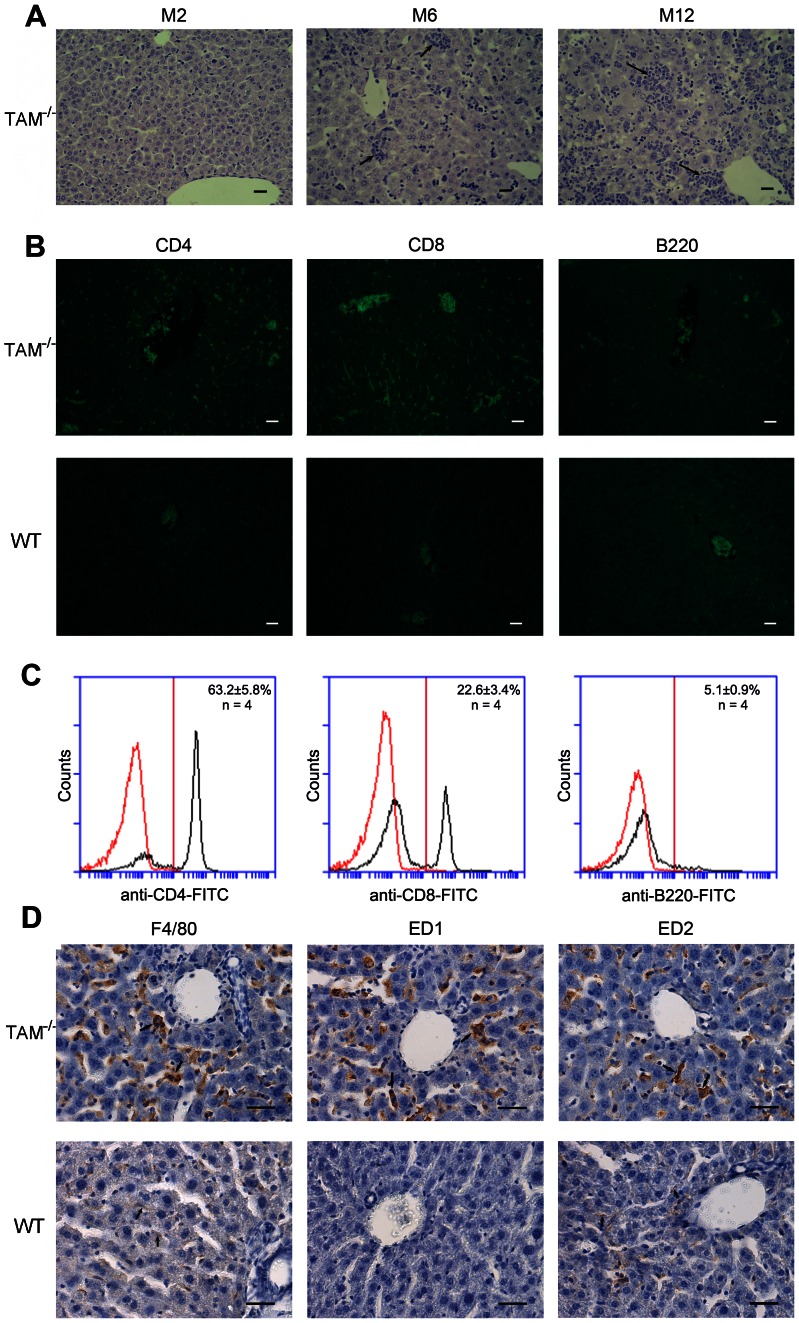
Histologic analysis of cellular infiltrations in the liver of TAM^−/−^ mice. (A) H&E staining of paraffin-embedded liver sections of TAM^−/−^ mice at the indicated ages. M1, M6 and M12 represent the liver sections of 1, 6 and 12 month-old mice respectively. Note a progression of cellular infiltrations in the liver as TAM^−/−^ mice aged. Arrows indicate focal infiltrates. (B) Characterization of infiltrated lymphocytes. Immunofluorescence was performed using FITC-conjugated antibodies against lymphocyte markers. Note that the infiltrated lymphocytes predominantly consist of CD4^+^ and CD8^+^ T cells. (C) Quantitatively analysis of lymphocytes. The cells were labeled with FITC-conjugated antibodies against CD4, CD8 and B220, and subsequently subject to flow cytometry. (D) Identification of macrophages. Immunohistochemistry was used for the identification of total macrophages (F4/80^+^), circulating macrophages (ED1^+^) and resident macrophages (ED2^+^). Note that the macrophages consist of resident and infiltrated cells in the liver of TAM^−/−^ mice, whereas only resident macrophages were observed in WT controls. Frozen liver sections of 10-month-old mice were used for the immune staining. Images are representatives of at least 5 mice. Scale bar  = 20 µm.

Histological analysis revealed an inflammatory phenotype characterized by immune cell infiltrations in the liver parenchymal regions of TAM^−/−^ mice (Figure 2A). This phenotype was more severe as mice aged. No apparent cell infiltration was observed in the liver of 2-month-old mice (M2). However, evident immune cell infiltrations were found in the liver of TAM^−/−^ mice at age 6 months (M6). The colonies of infiltrated cells grew larger as mice aged. At age 12 months (M12), infiltrated cell colonies were confluent, and most clusters of hepatocytes were disrupted. In contrast, no cellular infiltration was found in WT controls at any age from 1 to 12 months (data not shown).

The infiltrated cells contain macrophages and lymphocytes. The lymphocytes were confirmed by immunofluorescence staining, which consisted predominantly of CD4^+^ and CD8^+^ T cells, and relative few B cells (Figure 2B, upper panels). By contrast, much less lymphocytes were observed in the liver of age-matched WT mice (Figure 2B, lower panels). The three types of lymphocytes in the liver of TAM^−/−^ mice were quantitatively analyzed using flow cytometry (Figure 2C). The percentage of CD4^+^ cells was about 3-fold more than CD8^+^ cells. The macrophages in the parenchyma of TAM^−/−^ liver were determined by immunohistochemistry for F4/80. Abundant macrophages were detected in the TAM^−/−^ liver, which consisted of ED1^+^ circulating macrophages and ED2^+^ resident macrophages (Figure 2D, upper panels). In controls, only few F4/80^+^ and ED2^+^ cells were observed in the liver parenchymal regions of WT mice (Figure 2D, lower panels).

### Increased plasma IgG level and generation of autoantibodies in TAM^−/−^ mice

**Figure 3:**
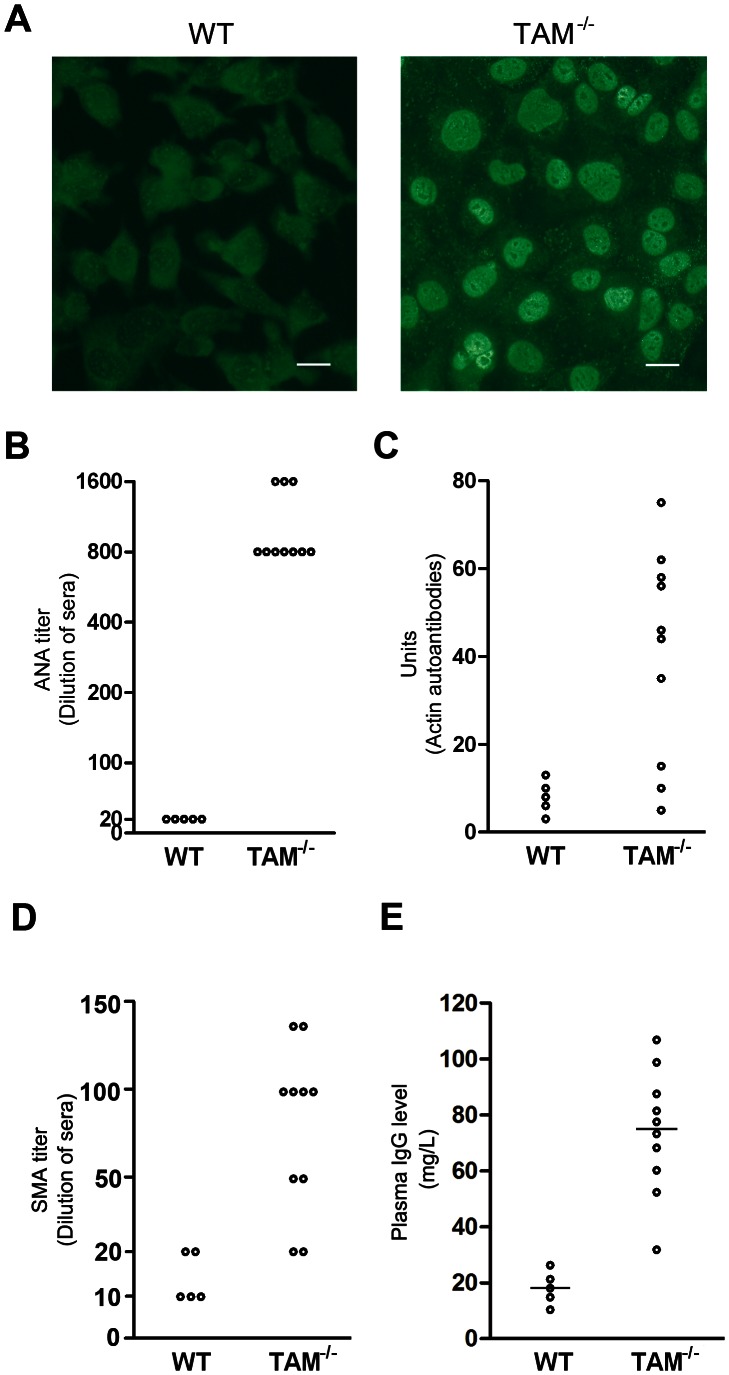
Autoantibodies diagnostic of AIH in sera from TAM^−/−^ **mice.** (A) Immunofluorescence on Hepa1-6 cells for detection of ANAs. The images are representative staining patterns using ×200 diluted sera of 12-month-old WT and TAM^−/−^ mice (n = 5 mice each genotype). Scale bar  = 40 µm. (B) The sera were diluted in series. Each circle indicates the maximum dilution of sera from individual mice, in which ANAs can be detectable. (C) Measurement of anti-actin autoantibody component of SMA using ELISA. Each circle indicates unit of anti-actin autoantibodies in sera of individual mice. (D) Titer of SMA. The microscope slides (Rat stomach) were stained using sera at the indicated dilutions. (E) Plasma IgG. The plasma were collected from 12-month-old mice. IgG levels were measured using ELISA.

Given that TAM^−/−^ mice exhibited phenotypes with several autoimmune diseases [25], we speculated that they may develop chronic AIH. Therefore, we examined characteristic serologic hallmarks of AIH, circulating autoantibodies and IgG level. Immunofluorescence staining on mouse hepatoma (Hepa1-6) cells showed the presence of ANA in the sera from TAM^−/−^ mice at age 12 months (Figure 3A). All sera obtained from 10 TAM^−/−^ female mice positively stained nuclei of Hepa1-6 cells at 1:800 times dilution (Figure 3B). The positive staining was even detected using 1,600 times diluted sera from three of these mice. In contrast, no ANA in the sera from WT mice was detected at >1∶20 dilution. The anti-actin autoantibody component of SMA was detected by ELISA in 7 of the 10 sera from TAM^−/−^ mice (Figure 3C). Titration analysis showed that SMA titers were more than 1∶20 in 8 of the 10 sera from TAM^−/−^ mice (Figure 3D). Plasma total IgG level in TAM^−/−^ mice at age 12 months was about 4-fold greater than that in WT mice (Figure 3E). These data indicate that TAM^−/−^ mice generate characteristic autoantibodies and high level of IgG.

### Expression of TAM RTKs and their ligands in the liver

**Figure 4:**
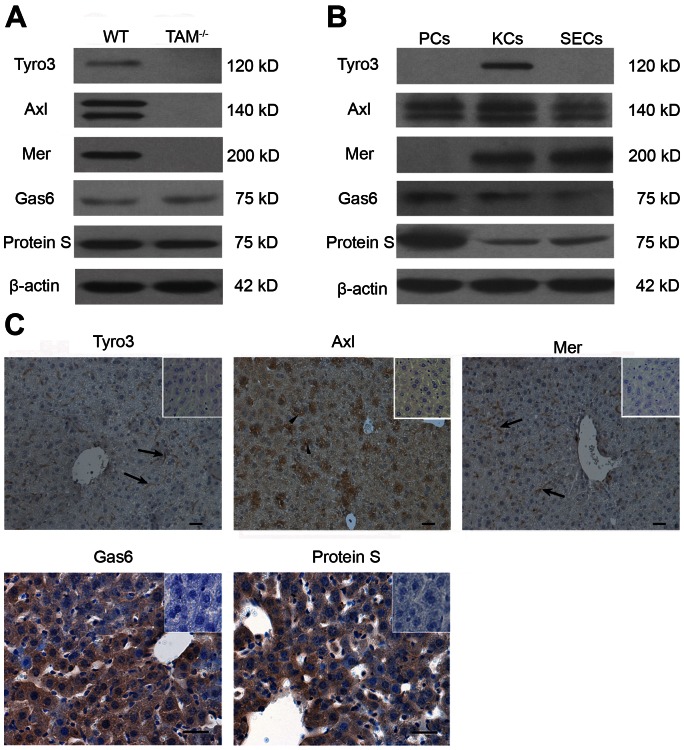
Expression of TAM RTKs, Gas6 and Protein S. (A) Western blot analyses of the liver lysates for the examination of TAM RTKs, Gas6 and Protein S. (B) Expression of TAM RTKs, Gas6 and Protein S in isolated liver cells: parenchymal cells (PCs), Kupffer cells(KCs) and sinusoidal endothelial cells (SECs). The primary cells were subjected to Western blotting. (C) Immunohistochemistry for the detection of TAM RTKs, Gas6 and Protein S. Arrowheads indicate PCs, and arrows indicate spindle-shaped sinusoidal cells corresponding to KCs and SECs. In negative controls (islets), sections were incubated with primary antibodies pre-incubated with an excess of blocking peptide. The livers of 15-week-old WT mice were used for the protein analyses. The images are representatives of at least three experiments. Scale bar  = 20 µm.

To understand the function of TAM RTKs in the liver, we examined the expression of TAM RTKs and their ligands (ProS and Gas6). All TAM RTKs and their ligands were detected in the liver of WT mice (Figure 4A). In contrast, TAM RTKs were absent in the liver of TAM^−/−^ mice, confirming that TAM alleles were null. To clarify the cell type-specific expression of TAM RTKs and ligands, we performed Western blotting of the isolated liver parenchymal cells (PCs) and nonparenchymal cells: Kupffer cells (KCs) and sinusoidal endothelial cells (SECs). As shown in Figure 4B, Tyro3 was only detected in KCs. However, Axl was abundantly expressed at a similar level in the three types of the liver cells. Mer was detected in KCs and SECs, but not in PCs. Gas6 and Protein S were prominently expressed in PCs, and relative weakly in KCs and SECs (Figure 4B). Immunohistochemistry confirmed that Tyro3 and Mer were localized in spindle-shaped sinusoidal cells, whereas Axl was evidently detected in some PCs (Figure 4C). Gas6 and Protein S were observed in all types of the liver cells based on immunohistochemical staining (Figure 4C).

### Upregulation of inflammatory cytokines in the liver of TAM^−/−^ mice

**Figure 5:**
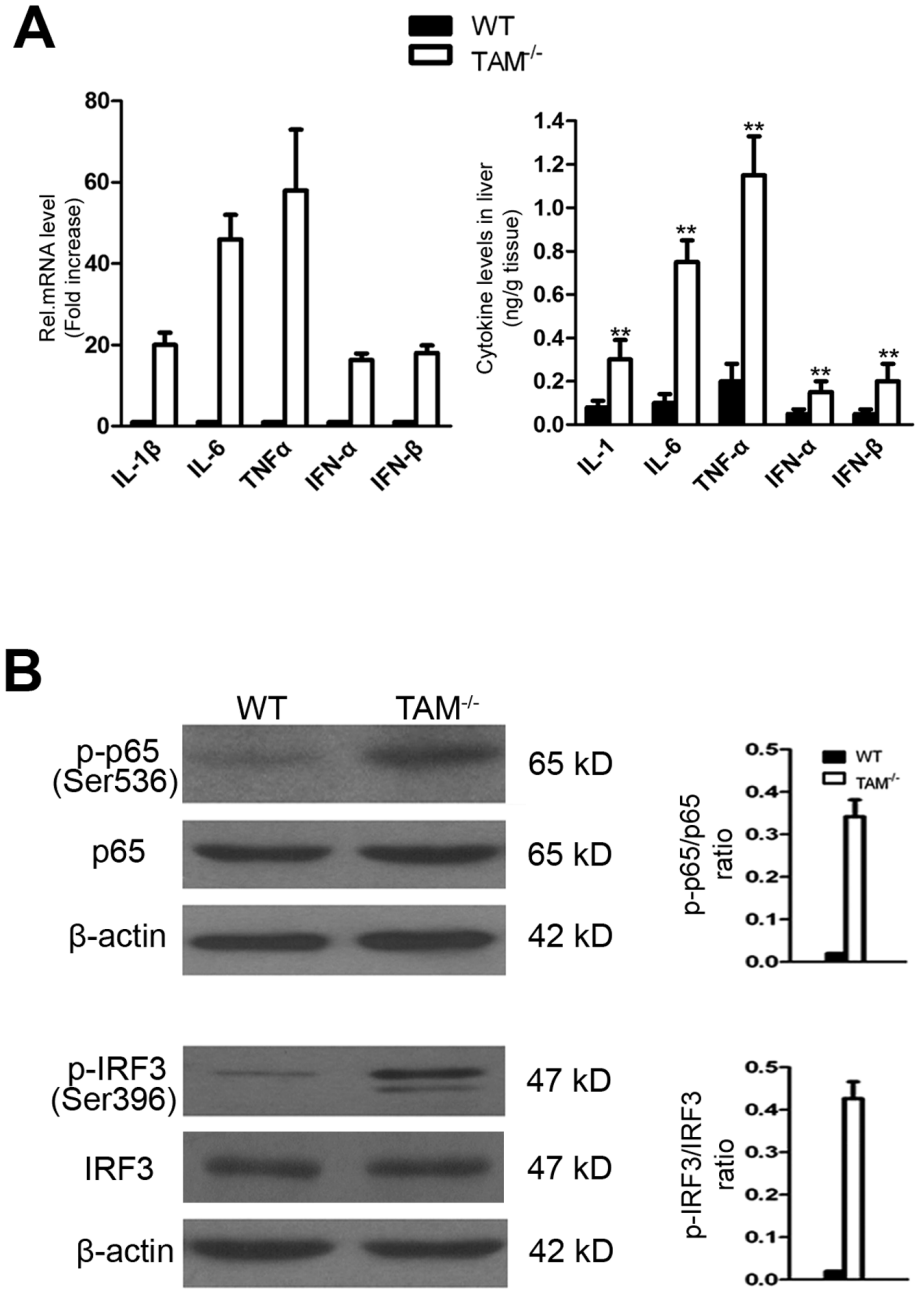
Activation of TLR signaling in the liver of TAM^−/−^ mice. (A) Expression of inflammatory cytokines. The mRNAs were analyzed by real-time RT-PCR (left panel) and the protein levels were determined by ELISA assay (right panel). Total RNAs were extracted from the liver and analyzed for the cytokine expression using RT-PCR, and cytokine levels in liver lysates were measured using ELISA. Data are mean ± SEM of three independent experiments. **P<0.01. (B) Analysis on the phosphorylation of NF-κB and IRF3. The liver lysates were used for immunoblots probed for phosphor-P65 (p-P65) and phosphor-IRF3 (p-IRF3).

Given that TLR-mediated inflammatory cytokine production is involved in the development of autoimmune liver diseases [12] and that TAM RTKs are inhibitors of inflammatory responses [28], we examined the expression of inflammatory cytokines in the liver of TAM^−/−^ mice. We found that, the inflammatory cytokines including interleukin (IL)-1β, IL-6, tumor necrosis factor alpha (TNF-α) and type 1 interferons (IFN-α and IFN-β) were dramatically upregulated in the liver of TAM^−/−^ mice (Figure 5A). These cytokines can be induced by the activation of nuclear factor (NF)-κB and interferon regulatory factor 3 (IRF3), leading to the induction of numerous inflammatory cytokines. Accordingly, NF-κB and IRF3 are activated in the liver of TAM^−/−^ mice as evident phosphorylation of NF-κB and IRF3 was observed (Figure 5B).

### Bone marrow-derived cells are critical in the liver damage

**Figure 6:**
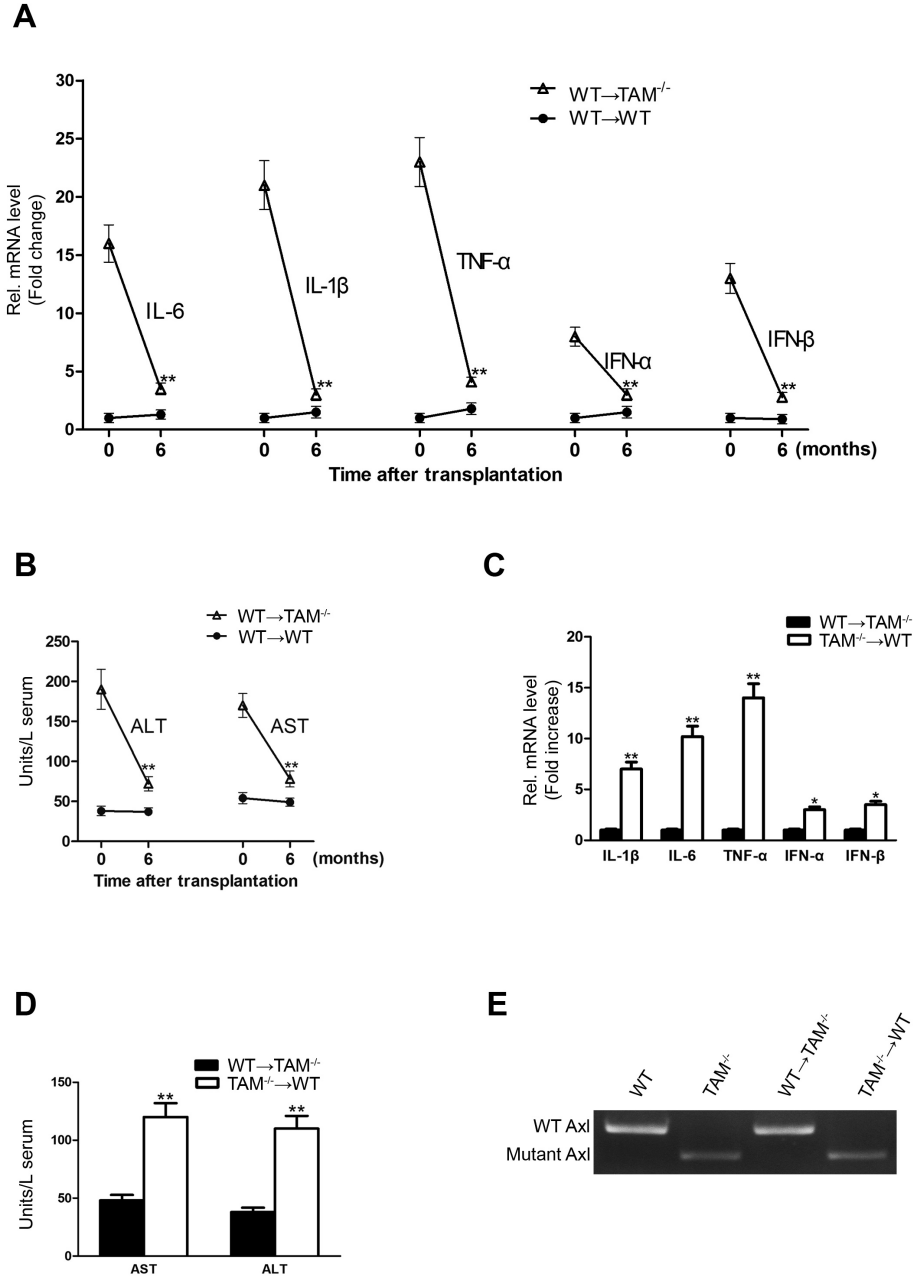
Bone marrow transplantation. Lethally irradiated 6-month-old mice were injected with bone marrow from 2-month-old donor mice through a tail vein. The engrafted mice were analyzed at 6 months after bone marrow transplantation. (A) Expression of inflammatory cytokines in the liver of WT→TAM^−/−^ mice. (B) Activities of serum ALT and AST in WT→TAM^−/−^ mice. (C) Cytokine expression in the liver of TAM^−/−^→WT mice. (D) Serum AST and ALT activities in the TAM^−/−^→WT mice. Total RNA was extracted from the liver, and the mRNAs of cytokines were determined by real-time RT-PCR. Serum AST and ALT activities were measured by enzymatic assay kits. (E) The expression of Axl in the circulating leukocytes in engrafted mice. Data are mean ± SEM (n = 10). **P<0.01.

To determine the contribution of infiltrated cells and resident liver cells to the liver damage in TAM^−/−^ mice, we performed bone marrow transplantation assays. At 6 months after transplantation, WT bone marrow cells transferred into irradiated TAM^−/−^ (WT→TAM^−/−^) mice resulted in dramatically down-regulation of inflammatory cytokine expression in the liver (Figure 6A). The liver damage was reduced in the engrafted mice, as serum ALT and AST activities in TAM^−/−^ recipients were significantly decreased (Figure 6B). By contrast, the transplantation of TAM^−/−^ bone marrows into WT (TAM^−/−^→WT) mice resulted in significant augmentation in inflammatory cytokine production in the liver and serum ALT/AST activities at 6 months after transplantation (Figure 6C, D). Notably, histological analysis revealed that cellular infiltrations were observed in the liver of TAM^−/−^→WT mice, but not in the liver of WT→TAM^−/−^ mice (data not shown). To confirm the origin of circulating cells, the genotype of peripheral leukocytes was examined by PCR. Amplification of mutant and wild-type Axl showed that the circulating leukocytes in engrafted mice were from donor mice (Figure 6E). The results suggest that bone marrow-derived cells lacking TAM RTKs are critical in the liver damage.

## Discussion

TAM RTKs are potent negative regulators of innate immunity and required for the optimal phagocytic clearance of apoptotic cells [29], [34], [35]. Mice lacking function of TAM RTKs display a profound dysregulation of the immune responses [25]. Here, we demonstrate that TAM^−/−^ mice develop progressively immune liver damage. This is the first report showing the liver inflammatory disease in the TAM^−/−^ mice.

The lack of a reliable animal model impedes the understanding the etiology of AIH. Several attempts have been made to develop animal models that can reflect the persistent liver damage occurring in human AIH. However, most models have only been successful in causing a transient form of liver damage [36], [37]. Our present study shows that TAM^−/−^ mice develop progressively liver inflammation and a persistent liver damage. Moreover, we demonstrated that TAM^−/−^ mice generate increased plasma IgG level, non-organ specific autoantibodies including ANA and SMA. These phenotypes are the characteristics of chronic AIH [37]. Notably, ANA and SMA in TAM^−/−^ mice could be secondary to an antigen driven response by the accumulation of apoptotic and necrotic cells in different tissues, because TAM receptor mutation impairs phagocytic clearance of apoptotic cells by macrophages [35].

The liver is constantly exposed to the microbial products from the enteric microflora through the portal circulation to the liver in physiological condition [38]. However, no obvious inflammation occurs under what would be pathologic condition in other tissues, due to “ liver tolerance ” [10]. The liver tolerance may arise from different mechanisms [39]. Immune damage of the liver requires the priming of liver-specific T cells, which then migrate into the liver to induce autoimmunity [40], [41]. Our present study showed that the immune cells including macrophages and T lymphocytes evidently infiltrate into the liver of TAM^−/−^ mice. Notably, more CD4^+^ vs CD8^+^ T cells were found, which correspond to previous observation in AIH [42]. A recent study indicated that activated liver-specific T cells alone were not sufficient to trigger the liver damage, and TLR3-driven inflammatory cytokine release was required to induce autoimmune liver disease [43]. The immune cell infiltrations and elevated inflammatory factors in the liver of TAM^−/−^ mice should be responsible for the liver damage. This speculation is also supported by the previous reports that IFN-α,IFN-β and TNF-α are involved in the pathogenesis of autoimmune diseases and that elevated TNF-α plays an essential role in the progression of liver injury [44], [45], [46]. Moreover, we observed augmentations in the expression of cell adhesion proteins including VCAM-1, ICAM-1 and E-selectin in the liver of TAM^−/−^ mice (data not shown), which should be responsible for the immune cell infiltrations.

The induction of inflammatory cytokines could result from activation of NF-κB and IRF3. We showed that NF-κB and IRF3 are activated in the liver of TAM^−/−^ mice. Upon pathogen stimulation, wild-type Kupffer cells and TAM^−/−^ peritoneal macrophages produce high levels of inflammatory cytokines [33], [47], [48]. Hepatic stellate cells (HSCs) express cell adhesion molecules (VCAM-1, ICAM-1 and E-selectin) in response to invading pathogens [49]. In addition, hepatic dendritic cells (DCs) and NK cells synthesize inflammatory factors and chemokines [50], [51]. Notably, these hepatic cells are less responsive to pathogen stimulation compared to those sentinel cells such as circulating DCs and macrophages [15]. Therefore, the upregulation of these inflammatory cytokines in the liver of TAM^−/−^ mice should be mainly attributable to the infiltrated immune cells. This speculation is supported by the literatures showing that TAM receptors inhibit peripheral DC and macrophage activation [25]. TAM RTKs were prominently expressed in non-parenchymal cells except that Axl was also detected in parenchymal hepatocytes. Recent findings revealed that TAM RTKs are potent inhibitors of TLR-triggered innate immune response in different types of cells [28], [52], [53]. Further, all hepatic cells express ProS and Gas6. It should be worthwhile to investigate whether ProS/Gas6-TAM signaling inhibits innate immune responses in the liver, thus contribute to the maintenance of the liver tolerance.

Although some resident liver cells express TAM RTKs, loss of these receptors in the resident liver cells is not sufficient for the liver damage. However, bone marrow-derived cells play a critical role in the liver damage. The inflammatory cytokine expression and the liver damage in WT→TAM^−/−^ mice were significantly reduced compared to TAM^−/−^ mice, and cellular infiltrations were not observed in the liver of these engrafted mice. By contrast, we observed the immune cell infiltrations, upregulation of inflammatory cytokines in the liver of TAM^−/−^→WT mice and the liver damage. These results suggest that the infiltrated cells lacking TAM RTKs are responsible for the cellular infiltrations, elevated inflammatory cytokines and liver damage. We speculate that loss of TAM RTKs in the immune cells promotes cellular infiltrations into the liver, and the infiltrated cells produced the elevated inflammatory cytokines, thus results in the liver damage.

In conclusion, the present study reveals that TAM RTKs play a critical role in maintaining the liver tolerance. Disruption of TAM function converts spontaneous inflammatory responses leading to chronic inflammatory liver damage. Data provide insights into the mechanisms underlying the liver tolerance.
